# GDF11 decreases bone mass by stimulating osteoclastogenesis and inhibiting osteoblast differentiation

**DOI:** 10.1038/ncomms12794

**Published:** 2016-09-22

**Authors:** Weiqing Liu, Liyan Zhou, Chenchen Zhou, Shiwen Zhang, Junjun Jing, Liang Xie, Ningyuan Sun, Xiaobo Duan, Wei Jing, Xing Liang, Hu Zhao, Ling Ye, Qianming Chen, Quan Yuan

**Affiliations:** 1 State Key Laboratory of Oral diseases, West China Hospital of Stomatology, Sichuan University, Chengdu 610041, China

## Abstract

Osteoporosis is an age-related disease that affects millions of people. Growth differentiation factor 11 (GDF11) is a secreted member of the transforming growth factor beta (TGF-β) superfamily. Deletion of *Gdf11* has been shown to result in a skeletal anterior–posterior patterning disorder. Here we show a role for GDF11 in bone remodelling. GDF11 treatment leads to bone loss in both young and aged mice. GDF11 inhibits osteoblast differentiation and also stimulates RANKL-induced osteoclastogenesis through Smad2/3 and c-Fos-dependent induction of *Nfatc1*. Injection of GDF11 impairs bone regeneration in mice and blocking GDF11 function prevents oestrogen-deficiency-induced bone loss and ameliorates age-related osteoporosis. Our data demonstrate that GDF11 is a previously unrecognized regulator of bone remodelling and suggest that GDF11 is a potential target for treatment of osteoporosis.

## Abstract (web summary)

GDF11 is related to myostatin yet has no known role in postnatal bone turnover. Here the authors show that recombinant GDF11 injection causes bone loss and impairs healing by driving osteoclastogenesis while inhibiting osteoblast differentiation, plus they show that anti-GDF11 Ab can inhibit bone loss in ovariectomy and ageing mouse models.

## Graphical abstract

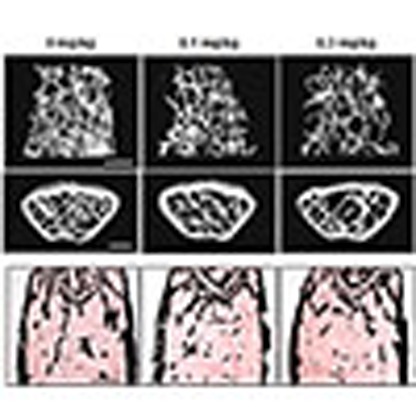

Osteoporosis is a skeletal disorder that affects millions of individuals, especially older and postmenopausal women^1^,^2^. The skeleton is continuously remodelled by a process in which pre-existing bone is removed by osteoclasts, specialized multinucleated cells derived from haematopoietic precursors, and rebuilt by osteoblasts derived from mesenchymal stem cells^3^,^4^,^5^. Bone loss occurs when the proper balance or bone formation and bone resorption is disrupted. Local cytokines and growth factors secreted by bone cells, as well as systemic hormones, have important roles in maintaining this critical balance^6^,^7^,^8^.

GDF11 is a secreted member of the transforming growth factor beta (TGF-β) superfamily^9^, several members of which have been implicated in regulating bone remodelling^10^. For example, TGF-β induces osteoclast formation^11^,^12^ and represses osteoblast differentiation^13^,^14^,^15^. Myostatin, which is highly homologous with GDF11 (ref. 9), was shown to induce osteoclastogenesis^16^. The direct role of GDF11 in bone development has been demonstrated in knockout mice that display skeletal patterning problems related to anterior–posterior positioning^17^,^18^. Repression of GDF11 function by transgenic overexpression of skeletal GDF11 propeptide results in an increase in bone mass^19^. GDF11 also regulates the differentiation of odontoblasts and repair of injured dentin^20^,^21^. However, the possible function of GDF11 in postnatal bone remodelling *in vivo* is unclear.

Some studies suggest that GDF11 acts as an anti-ageing factor^22^,^23^,^24^,^25^. Serum levels of GDF11 decline with age and injection of recombinant GDF11 (rGDF11) seems to reverse age-related dysfunction in brain, heart and skeletal muscle by rejuvenating stem cells^22^,^23^,^24^,^25^. However, other studies demonstrate opposite results, showing that GDF11 level does not change or increase with age^26^,^27^, and elevated GDF11 is a risk factor for age-related frailty and disease in humans^26^,^28^. Systemic administration of rGDF11 in mice inhibits skeletal muscle regeneration^27^ and fails to rescue age-related pathological cardiac hypertrophy^29^. These conflicting results led us to study whether GDF11 is associated with age-related bone loss. Here we show that injection of rGDF11 results in bone loss, as well as inhibition of bone regeneration in both young and aged mice. Importantly, the delivery of a GDF11 blocking antibody prevents oestrogen-deficiency-induced bone loss and ameliorates age-related osteoporosis, implying that GDF11 inhibition might be a potential therapeutic approach to prevent osteoporotic bone loss.

## Results

### GDF11 treatment leads to bone loss in young adult mice

**Figure 1:**
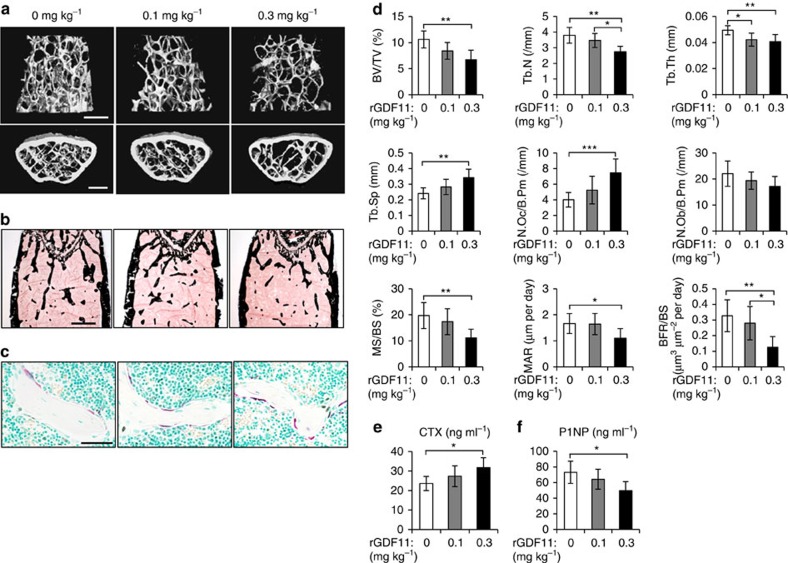
GDF11 treatment leads to bone loss in young adult mice. (**a**) Representative images of microCT reconstruction of distal femurs. Scale bar, 500 μm. (**b**) Von Kossa staining of undecalicified sections of femurs. Scale bar, 500 μm. (**c**) TRAP staining of femur sections from vehicle and rGDF11 treated mice. Scale bar, 50 μm. (**d**) Histomorphometric analysis of the metaphysis region of distal femurs. Results are shown as mean±s.d.; *n*=6–10; *P<0.05, **P<0.01 and ***P<0.001 by analysis of variance (ANOVA) with Tukey's *post hoc* test. (**e**,**f**) The serum levels of CTX and P1NP. Results are shown as mean±s.d.; *n*=6–10; *P<0.05 by ANOVA with Tukey's *post hoc* test.

We first examined the role of GDF11 in bone remodelling by administering young adult mice (9 week old) with daily intraperitoneal injections of rGDF11 (0.1 or 0.3 mg kg^−1^ body weight; #120-11, PeproTech, Rocky Hill, NJ) or vehicle (saline) for 6 weeks. MicroCT analysis of the secondary spongiosa of the distal femur metaphysis revealed that the trabecular bone volume (BV/TV) in mice treated with the higher dose of rGDF11 (0.3 mg kg^−1^) was significantly lower than in mice treated with vehicle (Fig. 1a,d). Mice given the lower dose of rGDF11 (0.1 mg kg^−1^) also showed a trend of decreased trabecular bone volume, although it was not statistically significant (*P*=0.078). Von Kossa staining further confirmed the loss of trabecular bone in high-dose rGDF11 mice (Fig. 1b). Histomorphometric analysis revealed a significant increase in osteoclast number (N.Oc/B.Pm) and a decrease in osteoblast number (N.Ob/B.Pm; Fig. 1c,d). Meanwhile, the mineral apposition rate (MAR) and bone formation rate (BFR/BS) in the high-dose rGDF11 mice were both significantly lower than the rates in vehicle-treated controls (Fig. 1d), indicating a reduction in bone formation. We also performed enzyme-linked immunosorbent assay (ELISA) to assess the serum markers for bone turnover. The serum levels of c-terminal telopeptides of collagen type I (CTX), a marker for bone resorption, were significantly higher in high-dose rGDF11 mice (Fig. 1e). In contrast, the serum levels of the bone formation marker P1NP were reduced (Fig. 1f).

### GDF11 treatment leads to bone loss in aged mice

**Figure 2:**
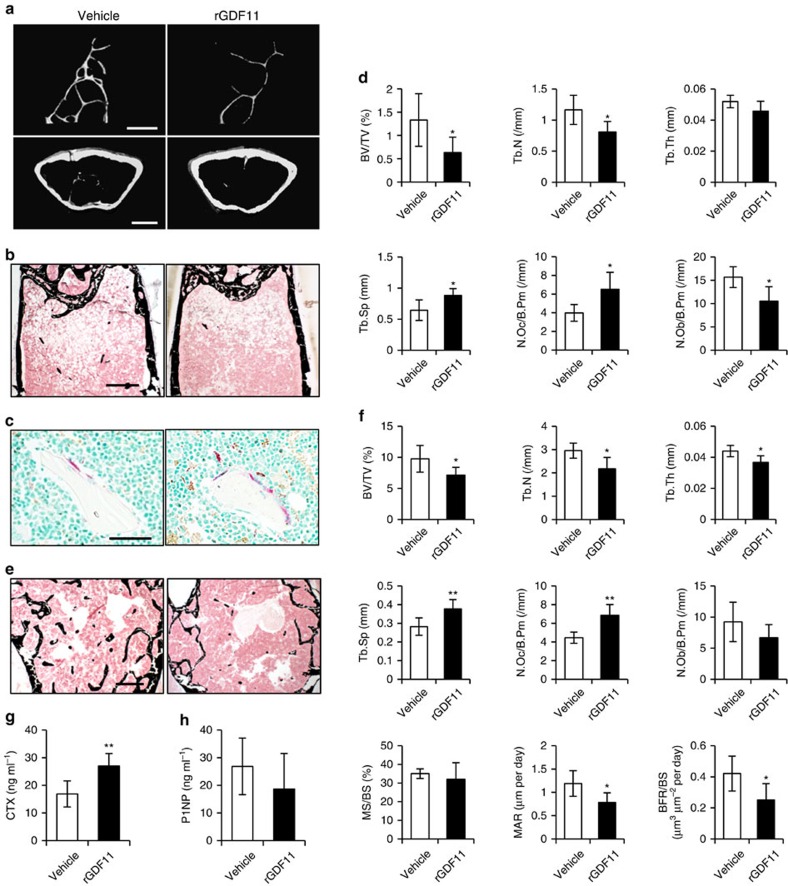
GDF11 treatment leads to bone loss in aged mice. (**a**) Representative images of microCT reconstruction of distal femurs. Scale bar, 500 μm. (**b**) Von Kossa staining of undecalicified sections of femurs. Scale bar, 500 μm. (**c**) TRAP staining of femur sections from vehicle and rGDF11 treated mice. Scale bar, 50 μm. (**d**) Histomorphometric analysis of the metaphysis region of distal femurs. Results are shown as mean±s.d.; *n*=6; *P<0.05 by *t* test. (**e**) Representative images of lumber 4 vertebrae stained with Von Kossa. Scale bar, 500 μm. (**f**) Histomorphometric analysis of the trabecular bone in vertebrae. Results are shown as mean±s.d.; *n*=6; *P<0.05 and **P<0.01 by *t* test. (**g**,**h**) The serum levels of CTX and P1NP. Results are shown as mean±s.d.; *n*=6; **P<0.01 by *t* test.

Given the controversial role of GDF11 in aging pathologies, we also investigated the effect of rGDF11 in aged mice (18 month old) by daily intraperitoneal injections of 0.3 mg kg^−1^ rGDF11 for 6 weeks. Consistent with young adult mice, we found that rGDF11 administration led to trabecular bone loss in the distal femur metaphysis of aged animals, with increased osteoclast number (N.Oc/B.Pm) and decreased osteoblast number (N.Ob/B.Pm) (Fig. 2a–d). There is little trabecular bone left in the femur metaphysis of aged mice, so we evaluated the L4 vertebrae and observed similar results (Fig. 2e,f). In addition, the serum levels of CTX were also significantly elevated in rGDF11 treated mice (Fig. 2g).

Previous studies showed that rGDF11 may rejuvenate muscle stem cells^24^. We therefore investigated whether rGDF11 (0.3 mg kg^−1^) administration might have a similar effect on bone marrow stem cells (BMSCs). However, we observed that the percentage of BMSCs, as determined by flow cytometry, did not change in either young or aged mice after daily treatments for 6 weeks (Supplementary Fig. 1a,b). We compared colony formation in these sorted cells and found no significant differences between cells from treated and control mice (Supplementary Fig. 1c,d). Moreover, the rGDF11 treatment had no apparent effect on the proliferation of these sorted cells (Supplementary Fig. 1e).

### GDF11 stimulates RANKL-induced osteoclastogenesis

**Figure 3:**
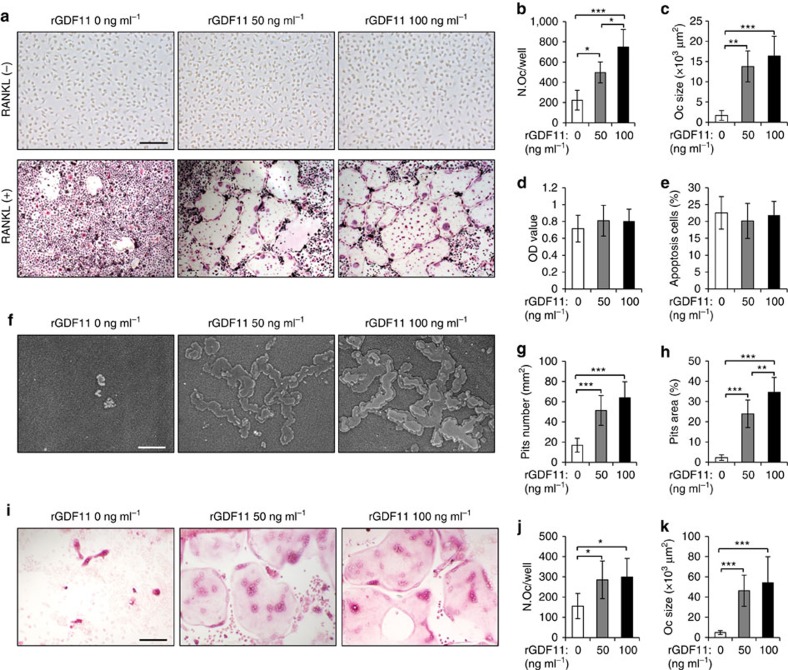
GDF11 stimulates RANKL-induced osteoclastogenesis. (**a**) Representative TRAP staining of the BMMs after 4 days of osteoclast differentiation. 100 ng ml^−1^ M-CSF was present in all settings. Scale bar, 100 μm. (**b**,**c**) Osteoclast number and size. TRAP-positive cells with at least three nuclei were counted as osteoclasts. Results are shown as mean±s.d.; *n*=9; *P<0.05, **P<0.01 and ***P<0.001 by analysis of variance (ANOVA) with Tukey's *post hoc* test. (**d**,**e**) Cell proliferation and apoptosis assay. BMMs were cultured in the presence of 100 ng ml^−1^ M-CSF and 50 ng ml^−1^ RANKL with indicated amount of rGDF11 for 2 days. Results are shown as mean±s.d.; *n*=8. (**f**) Representative images of resorption pits on Osseo Assay surface after 7 days of culture. Scale bar, 100 μm. (**g**,**h**) Resorption pits number and resorption area. Results are shown as mean±s.d.; *n*=8; **P<0.01 and ***P<0.001 by ANOVA with Tukey's *post hoc* test. (**i**) Representative images of osteoclasts in co-cultures with osteoblasts in presence of 1,25(OH)_2_D_3_ and PGE_2_ with indicated amount of rGDF11 for 5 days. Scale bar, 100 μm. (**j**,**k**) Osteoclast number and size for the co-cultures. Results are shown as mean±s.d.; *n*=8. *P<0.05 and ***P<0.001 by ANOVA with Tukey's *post hoc* test.

We next assessed the direct contribution of GDF11 to osteoclast-mediated bone resorption. We analysed osteoclast differentiation *in vitro* and found that rGDF11 alone was not able to induce osteoclastogenesis of bone marrow-derived macrophages (BMMs; Fig. 3a, upper panel). We therefore supplemented medium containing receptor activator of nuclear factor kappa-B ligand (RANKL) with rGDF11. The presence of either 50 ng ml^−1^ or 100 ng ml^−1^ rGDF11 in the medium significantly stimulated osteoclast differentiation, as evidenced by an increase of the number of tartrate-resistant acid phosphatase (TRAP) positive multinucleated cells after 4 days of treatment (Fig. 3a lower panel, b). Notably, rGDF11 treatment also resulted in very large osteoclasts with a huge cytoplasmatic compartment, which indicates that GDF11 stimulated osteoclast maturation (Fig. 3c). On the contrary, only a few small osteoclasts were generated in control media during this short test period. The presence of rGDF11 did not significantly affect the proliferation or apoptosis of BMMs (Fig. 3d,e). We next performed resorption pit analyses using the Osseo Assay plate (Corning) and observed a larger number of resorption pits and a larger area overall in the presence of rGDF11 compared with RANKL treatment alone (Fig. 3f–h). Finally, we examined osteoclast formation in a co-culture of BMMs and calvarial osteoblasts to mimic the *in vivo* environment. The presence of rGDF11 led to an obvious increase of the number and size of TRAP-positive multinucleated cells (Fig. 2i–k).

**Figure 4:**
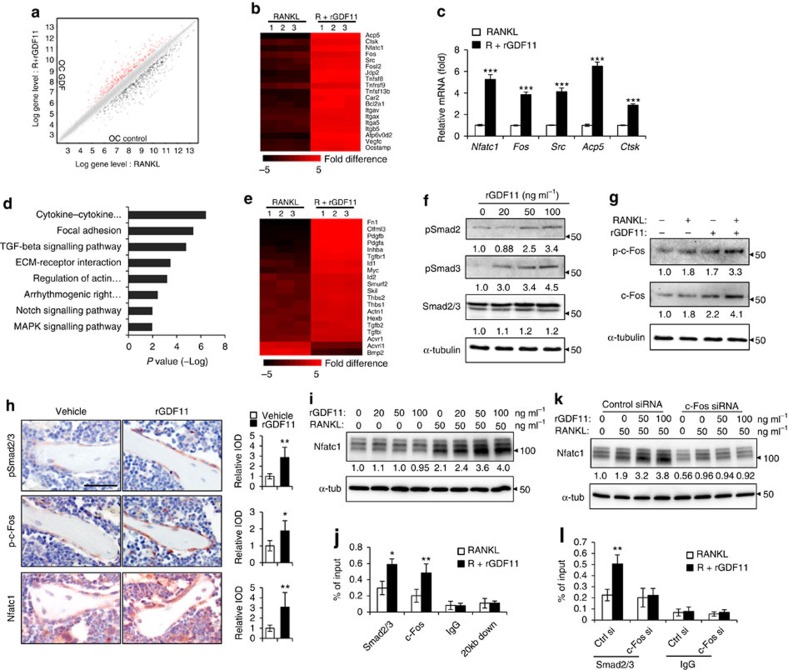
GDF11 activates Smad2/3-dependent TGF-β pathway. (**a**) Gene expression levels of the BMMs stimulated without or with 100 ng ml^−1^ rGDF11 for 24 h (three biological replicates per group). 467 genes (red) were upregulated and 679 genes (black) were downregulated. 100 ng ml^−1^ M-CSF was present in all settings. (**b**) Heatmap of the osteoclastogenesis associated genes. (**c**) Quantitative RT-PCR confirmed the increased expression of osteoclast key marker genes. Results are shown as mean±s.d.; *n*=3. ***P<0.001 by *t* test. (**d**) KEGG pathway analysis indicated the altered function of TGF-β pathway. (**e**) Heatmap of the TGF-β pathway associated genes. (**f**) Western blot analysis indicated that rGDF11 stimulated the phosphorylation of Smad2/3 in BMMs in 30 min. (**g**) Western blot analysis demonstrated that rGDF11 amplified the RANKL-induced expression of c-Fos. BMMs were starved overnight and then treated for 4 h. (**h**) Representative images of immunohistochemical staining. rGDF11 injections increased the phosphorylation of Smad2/3 and c-Fos, as well as the expression of Nfatc1 *in vivo*. Femurs were collected ∼2 h after the last injection of rGDF11. Scale bar, 50 μm. (**i**) Western blot analysis indicated that rGDF11 stimulated the RANKL-induced expression of Nfatc1. BMMs were treated for 2 days. (**j**) ChIP assay revealed that rGDF11 induced the co-occupancy of Smad2/3 and c-Fos to the binding region of *Nfatc1*. Results are shown as mean±s.d.; *n*=3. *P<0.05 and **P<0.01 by *t* test. (**k**) Western blot analysis of Nfatc1. Depletion of c-Fos eliminated the rGDF11 induced expression of Nfatc1. (**l**) ChIP assay. Depletion of c-Fos abolished rGDF11 triggered binding of Smad2/3 to *Nfatc1*. Results are shown as mean±s.d.; *n*=3. **P<0.01 by *t* test.

We further elucidated the molecular mechanism by performing a microarray analysis. Compared with RANKL alone, rGDF11 supplementation increased the expression of 467 genes (Fig. 4a), including *Nfactc1*, the master transcriptional factor of osteoclast differentiation, as well as other key marker genes, such as *Fos*, *Src*, *Acp5* and *Ctsk* (Fig. 4b,c). Notably, a KEGG pathway analysis also indicated that GDF11 upregulated the expression of genes associated with TGF-β pathway (Fig. 4d,e). We also observed that rGDF11 was capable of activating the phosphorylation of Smad2/3 in mouse BMMs *in vitro* (Fig. 4f, and Supplementary Fig. 4 for uncropped images). This is consistent with previous findings in myotubes and myocardium^25^,^27^. In addition, rGDF11 administration amplified the RANKL-induced expression of c-Fos (Fig. 3g). Immunohistochemical staining of the bone sections demonstrated that the numbers of pSmad2/3 and p-c-Fos-positive cells were increased after rGDF11 injections (Fig. 3h). A recent study showed that Smad2/3 cooperates with c-Fos to regulate the expression of *Nfatc1* during osteoclastogenesis^30^. We observed increased expression of Nfatc1 upon rGDF11 treatment (Fig. 4b,c,h,i), so we performed a ChIP assay and found that rGDF11 significantly induced the recruitment of both Smad2/3 and c-Fos to the binding region of *Nfatc1* compared with RANKL treatment alone (Fig. 4j). We further investigated whether this effect is c-Fos dependent by performing loss of function experiments using c-Fos short interfering RNA (siRNA). Depletion of c-Fos eliminated the rGDF11 induced expression of Nfatc1 (Fig. 4k), and completely abolished the triggered binding of Smad2/3 to *Nfatc1* (Fig. 4l). These data, taken together, demonstrated that the effect of GDF11 on osteoclastogenesis is mediated by a Smad2/3 and c-Fos dependent increase in the transcription of *Nfatc1*.

### GDF11 inhibits osteoblast differentiation

**Figure 5:**
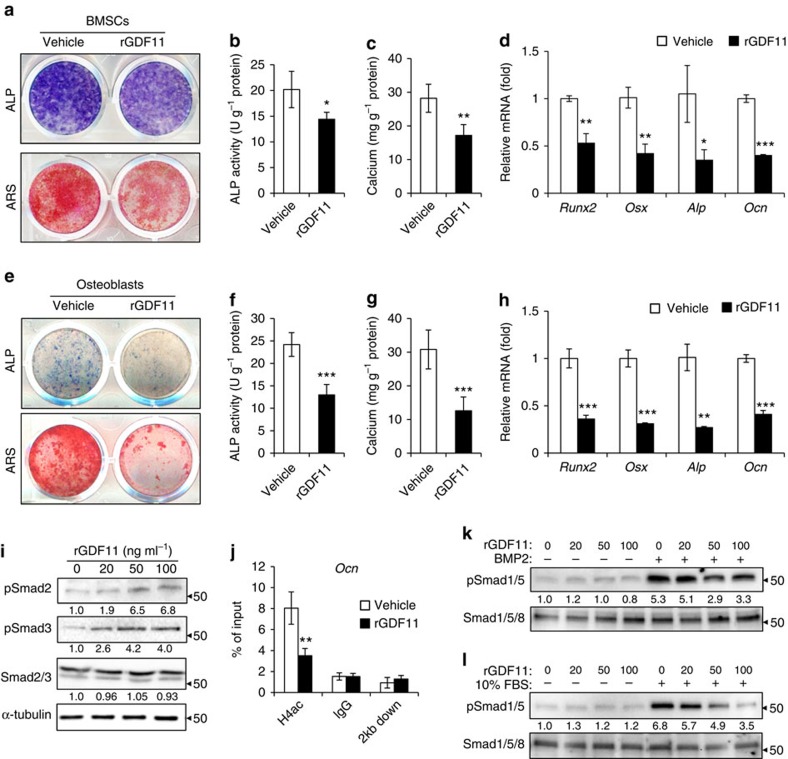
GDF11 inhibits osteoblast differentiation. (**a**) Representative images of ALP staining and Alizarin Red S (ARS) staining of BMSCs. (**b**,**c**) Quantitative analyses of the ALP activity and calcium mineralization in BMSCs. Results are shown as mean±s.d.; *n*=5; *P<0.05 and **P<0.01 by *t* test. (**d**) Quantitative RT-PCR revealed reduced messenger RNA expression of *Runx2*, *Osx*, *Alp* and *Ocn* in rGDF11 treated BMSCs. Results are shown as mean±s.d.; *n*=5; *P<0.05, **P<0.01 and ***P<0.001 by *t* test. (**e**) Representative images of ALP and ARS staining of primary calvarial osteoblasts. (**f**,**g**) Quantitative analyses of the ALP activity and calcium mineralization in osteoblasts. Results are shown as mean±s.d.; *n*=5; ***P<0.001 by *t* test. (**h**) Quantitative RT-PCR demonstrated that rGDF11 inhibited messenger RNA expression of *Runx2*, *Osx*, *Alp* and *Ocn* in osteoblasts. *n*=3. Results are shown as mean±s.d.; *n*=5; **P<0.01 and ***P<0.001 by *t* test. (**i**) Western blot indicated that rGDF11 stimulated the phosphorylation of Smad2/3 in osteoblasts. (**j**) ChIP assay revealed that rGDF11 reduced the abundance of acetylated histone H4 (H4ac) on the promoter of *Ocn*. Results are shown as mean±s.d.; *n*=3; **P<0.01 by *t* test. (**k**,**l**) Western blot analysis of pSmad1/5. The presence of rGDF11 attenuated BMP2 or foetal bovine serum induced phosphorylation of Smad1/5. Osteoblasts were starved overnight and then treated for 30 min.

We next investigated the effect of rGDF11 on osteoblast differentiation *in vitro*. BMSCs were cultured in osteogenic differentiation medium supplemented with or without rGDF11. The presence of rGDF11 (100 ng ml^−1^) significantly inhibited the osteogenic potential, as evidenced by alkaline phosphatase (ALP) staining and Alizarin Red S (ARS) staining (Fig. 5a). Quantitative analyses confirmed decreases in ALP activity (Fig. 5b) and calcium mineralization (Fig. 5c). Furthermore, quantitative PCR with reverse transcription (RT-PCR) revealed a reduction in messenger RNA expression of the master osteogenic transcription factors *Runx2*, as well as *Osx* (*Osterix*), *Alp* and *Ocn* (*Osteocalcin*) in rGDF11 treated BMSCs compared with controls (Fig. 5d). We also isolated primary calvarial osteoblasts for treatment with rGDF11. A similar, and sometimes an even more severe, inhibition of osteoblast differentiation was observed compared with the BMSCs (Fig. 5e–h). rGDF11 slightly increased the proliferation of both BMSCs and osteoblasts (Supplementary Fig. 2a,b).

We then determined whether GDF11 has a similar regulatory effect on the differentiation of osteoblasts via the TGF-β pathway. Western blot showed that rGDF11 did stimulate the phosphorylation of Smad2/3 in primary osteoblasts (Fig. 5i). We found that rGDF11 inhibited the expression of *Runx2* during osteogenic differentiation (Fig. 5d,h), which is in accordance with previous reports that the activation of Smad3 decreases the expression of Runx2 and represses its function by inducing histone H4 deacetylation at the osteocalcin promoter^15^,^31^. In addition, the ChIP assay demonstrated that rGDF11 significantly decreased the abundance of acetylated histone H4 (H4ac) at the Runx2 binding site of the osteocalcin promoter (Fig. 5j).

Activation of TGF-β may counteract BMP signalling^32^,^33^, which is critical for osteoblast differentiation^10^. Therefore we evaluated the effect of rGDF11 on BMP signalling. Interestingly, we found that rGDF11 attenuated BMP2 (100 ng ml^−1^, #120-02C, PeproTech, Rocky Hill, NJ) or foetal bovine serum (10%) induced phosphorylation of Smad1/5 in osteoblasts (Fig. 5k,l).

### GDF11 impairs the regeneration of bone defects

**Figure 6:**
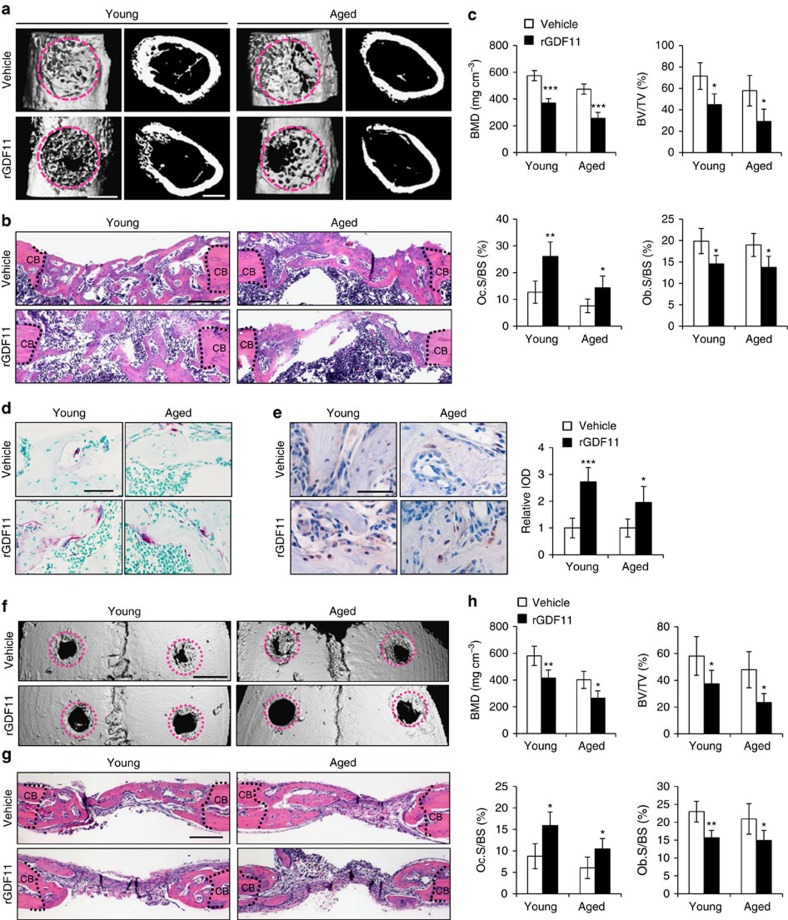
GDF11 impairs regeneration of bone defects. (**a**) Representative images of microCT reconstruction of femoral cortical bone defects. The red dotted lines indicate the position of the original defect margin. Scale bar, 500 μm. (**b**) H&E staining of femoral cortical bone defects. The black dotted lines indicate the position of the original defect margin. Abbreviation: CB, cortical bone. Scale bar, 200 μm. (**c**) Bone mineral density (BMD) and histomorphometric analysis of the regenerated bone in femoral cortical gaps. Results are shown as mean±s.d. The mean values of the bilateral defects of each mouse were counted as individual data points (*n*=5). *P<0.05, **P<0.01 and ***P<0.001 by *t* test. (**d**) Representative images of TRAP staining of the regenerated bone in femoral cortical gaps. Scale bar, 50 μm. (**e**) Representative images and quantification of immunohistochemical staining of pSmad2/3. rGDF11 injections increased the phosphorylation of Smad2/3. Scale bar, 50 μm. (**f**) MicroCT reconstruction of the calvarial defects. The red dotted lines indicate the position of the original defect margin. Scale bar, 1 mm. (**g**) H&E staining of the calvarial defects. The dotted lines indicate the position of the original defect margin. CB, cortical bone. Scale bar, 200 μm. (**h**) Bone mineral density (BMD) and histomorphometric analysis of the regenerated bone in calvarial defects. Results are shown as mean±s.d. The mean values of the bilateral defects of each mouse were counted as individual data points (*n*=5). *P<0.05 and **P<0.01 by *t* test.

We further investigated the effect of rGDF11 in a bone regeneration model of femoral cortical bone defects induced by drill hole injury^34^. Young and aged mice were administered with rGDF11 (0.3 mg kg^−1^) or vehicle (saline) for 2 weeks before the surgeries, and rGDF11 was administered daily throughout the study. Mice were necropsied 2 weeks after surgeries. MicroCT and histological analyses consistently showed that the cortical gaps were almost completely bridged in both young and aged vehicle mice, while those of rGDF11 treated ones were only partially filled (Fig. 6a,b). The volume (BV/TV) and density (BMD) of the mineralized callus of rGDF11 group were significantly lower when compared with the vehicle group (Fig. 6d). The osteoclast surfaces (Oc.S/BS) were largely elevated while osteoblast surfaces (Ob.S/BS) diminished (Fig. 6c,d). Immunohistochemical staining revealed more intensive pSmad2/3 signals in the rGDF11-treated group (Fig. 6e).

Next, we sought to determine whether GDF11 could have the same effect on bone regeneration of subcritical-sized calvarial defects. Two 1.0 mm bone defects were generated symmetrically across the sagittal suture. Only a small volume of newly formed bone was observed around the margin of this defect in the rGDF11 treated group (Fig. 6f,g). Moreover, the BV/TV and BMD were significantly lower than those in the vehicle group (Fig. 6h).

### Inhibition of GDF11 prevents OVX-induced bone loss

**Figure 7:**
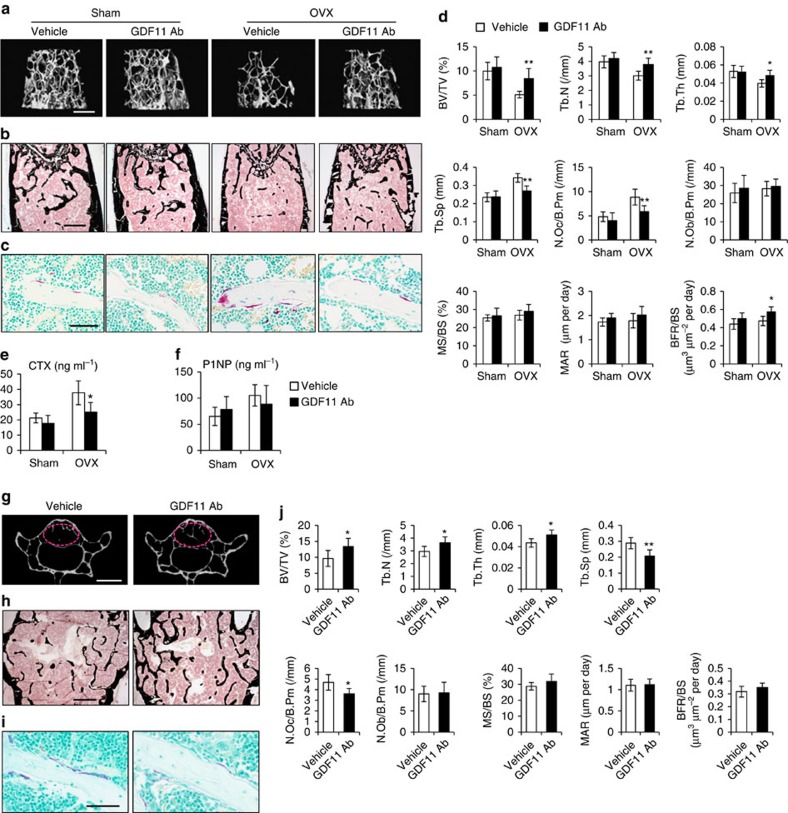
Inhibition of GDF11 prevents bone loss. (**a**) MicroCT reconstruction of trabecular bone from distal femurs. Scale bar, 500 μm. (**b**) Von Kossa staining of undecalicified sections of femurs. Scale bar, 500 μm. (**c**) TRAP staining of femur sections. Scale bar, 50 μm. (**d**) Histomorphometric analysis of the metaphysis region of distal femurs. Results are shown as mean±s.d.; *n*=6; *P<0.05 and **P<0.01 by *t* test. (**e**,**f**) The serum levels of CTX and P1NP. Results are shown as mean±s.d.; *n*=6; *P<0.05 by *t* test. (**g**) Representative images of microCT scanning of lumber 4 vertebrae. The red circles indicate the region of interest (ROI). Scale bar, 1 mm. (**h**) Von Kossa staining of undecalicified sections of lumber 4 vertebrae. Scale bar, 500 μm. (**i**) TRAP staining of vertebrae sections. Scale bar, 50 μm. (**j**) Histomorphometric analysis of the trabecular bone in lumber four vertebrae. Results are shown as mean±s.d.; *n*=6; *P<0.05 and **P<0.01 by *t* test.

Oestrogen deficiency in postmenopausal females leads to an imbalance between bone formation and bone resorption, which subsequently results in net bone loss and osteoporosis. Our results show that GDF11 plays a role in stimulating osteoclastogenesis and in inhibiting osteogenesis, so we hypothesized that inhibiting GDF11 could prevent oestrogen-deficiency-induced bone loss. To this end, sham and ovariectomized (OVX) mice were treated by intraperitoneal injections (100 μg per mouse per time, twice a week) of GDF11 antibody (Ab; MAB19581, clone #743833, R&D, Minneapolis, MN) starting 2 days after the OVX procedure and continuing for 6 weeks. GDF11 is highly related to myostatin (GDF8) and shares 90% homology in the mature active regions^9^. So we first performed western blot analysis to confirm that the batch of antibody we used in this experiment binds specifically to GDF11 without any cross-reaction with myostatin (Supplementary Fig. 3a,b). MicroCT analysis of the distal femur metaphysis revealed that the OVX procedure resulted in a nearly 50% bone loss in vehicle-treated mice (Fig. 7a,d). In contrast, the effect was significantly diminished in Ab-treated animals, where only a 21% bone loss occurred during the observation period (Fig. 7a,d). More importantly, the expected increases in osteoclast number (N. Oc/B.Pm) following OVX were significantly suppressed by GDF11 Ab administration (Fig. 7c,d). No differences were observed in the sham-operated mice treated with GDF11 Ab or vehicle (Fig. 7a–d). We also measured serum markers for bone turnover and found that the levels of CTX, a marker for bone resorption, were significantly reduced in GDF11 Ab-treated mice (Fig. 7e).

### Inhibition of GDF11 ameliorates age-related osteoporosis

Based on these first observations, we further explored the therapeutic potential of GDF11 Ab for age-related osteoporosis. 18-month-old female mice were given intraperitoneal injections of GDF11 Ab (100 μg per mouse per time, twice a week) for 4 weeks. MicroCT and histomorphometric analyses of the L4 vertebrae showed that treatment with the GDF11 Ab significantly improved the trabecular bone volume (BV/TV; Fig. 7g,h,j). It also increased the trabecular number (Tb.N) and thickness (Tb.Th), while reducing the trabecular separation (Tb.Sp; Fig. 7j). Moreover, the osteoclast number (N.Oc/B.Pm) was significantly suppressed by GDF11 Ab administration (Fig. 7i,j). Taken together, our results suggest that GDF11 inhibition is a potential therapeutic approach for postmenopausal bone loss and age-related osteoporosis.

## Discussion

Osteoporosis is an age-related disease that affects millions of people^1^,^2^. Aging populations around the world have fuelled continuing interest among biomedical researchers in finding anti-aging factors^35^,^36^. It has recently been suggested that GDF11 is one of the long-sought rejuvenation agents^22^,^23^,^24^,^37^. Lee's group first reported that circulating levels of GDF11 decrease with age and restoration of more youthful levels of systemic GDF11 appears to reverse age-related cardiac hypertrophy^22^. Later related studies suggested that injection of rGDF11 rejuvenates skeletal, muscle and brain function in aged mice^23^,^24^. A clinical study also suggested that higher GDF11/8 levels are associated with a lower risk of cardiovascular events and death in patients with stable ischaemic heart disease^38^. However, these observations were recently challenged^26^,^27^,^28^,^29^,^39^,^40^,^41^,^42^,^43^. Egerman *et al*.^27^ demonstrated that circulating GDF11 increases with age and systematic administration of rGDF11 actually inhibits muscle regeneration. Others also reported that GDF11 lacks an anti-hypertrophic effect *in vitro* and restoring GDF11 in old mice has no effect on cardiac structure or function^29^. In this study, we injected both young and aged mice with rGDF11, and did not observe any significant change in the pool of BMSCs in bone marrow or their ability for colony formation. Most recently, Schafer *et al*.^26^ showed that in humans, GDF11 levels do not change with age but are associated with comorbidity and frailty. In addition, Lee's group^25^ verified their previous reports that serum GDF11/8 levels decline with age in multiple mammalian species. Their results indicated that the increase in GDF11 levels, as reported by Egerman *et al*.^27^, is due to a cross-reaction of the GDF8/GDF11 antibody (Abcam) with immunoglobulin. This issue might be resolved with experiments performed parallel using the R&D GDF11 antibody. Others^27^,^29^ and we have verified that this antibody specifically detects GDF11 without cross-reaction with myostatin.

In this study, we first reported that rGDF11 treatment decreases bone mass that results from the uncoupling of bone resorption with bone formation. Previously, Li *et al*.^44^ generated transgenic mice that over-express the GDF11 propeptide under the control of a bone-specific regulatory element. The GDF11 propeptide forms a latent complex with GDF11 to antagonize GDF11 activity^45^, so this mouse might be considered as a model for repression of GDF11 function. Indeed, these transgenic mice exhibited an abnormal transformation of vertebral formation^44^. More importantly, these mice also showed a significant increase in bone mass^19^, which supports our finding that GDF11 induces bone loss. Recently, Zhang *et al*.^46^ reported a controversial finding that GDF11 induces osteoblast differentiation. However, we could not reproduce their results using rGDF11 from the same manufacturer and the same methods as they described. The conflicting findings may be caused by different batches of GDF11 from the manufacturer. Cells from different sources may be another possibility, but Zhang *et al*. did not describe the details of cell collection.

It should be noted that we could not fully exclude an indirect effect on bone mass caused by the systematic application of rGDF11. Recently, accumulating evidence suggests that GDF11 treatment may negatively affect the skeletal muscle^27^,^40^,^42^, although deletion of *Gdf11* specifically in skeletal muscle does not affect muscle size, fibre number or fibre type^47^. Given the role of muscle to bone crosstalk^48^,^49^,^50^, GDF11 treatment may also regulate bone mass indirectly by affecting skeletal muscle. In addition, we still lack direct genetic evidence for the role of GDF11 in postnatal bone remodelling. *Gdf11* knockout mice die shortly after birth and none of the currently available knockout or transgenic mouse models could avoid the effect of losing GDF11 during prenatal development^17^,^44^,^51^,^52^. A better approach is to generate the inducible knockout or overexpression mouse models for future studies.

GDF11 is highly related to myostatin (GDF8) with 90% homology in the mature active regions^9^. These two proteins also have redundant functions in regulating skeletal patterning in mice^47^. Here we show that GDF11 inhibits osteoblast differentiation and stimulates RANKL-induced osteoclastogenesis, which is also similar to the functions of myostatin. Previous studies showed that loss of myostatin increases osteogenic differentiation in BMSCs and that myostatin-null mice exhibit improved bone mass and bone mineral density^53^,^54^,^55^. Recently, Dankbar *et al*.^16^ demonstrated that myostatin directly regulates osteoclast differentiation and its inhibition reduces inflammatory joint destruction in mice. The effects of GDF11 and myostatin on osteoclasts also appear similar as both stimulate RANKL-mediated osteoclastogenesis through the Smad2/3-dependent TGF-β pathway. GDF11 rapidly induces the phosphorylation of Smad2/3 and c-Fos, both *in vitro* and *in vivo*, and increases the transcription of *Nfatc1*.

In this study, we demonstrate that GDF11 is a previously unrecognized regulator of bone remodelling. The bone loss induced by rGDF11 injections likely represents a pharmacological effect of rGDF11, given their low endogenous levels in mice. However, we did show that blocking of endogenous GDF11 function prevents oestrogen-deficiency-induced bone loss and age-related osteoporosis, indicating GDF11 does have effects on bone metabolism physiologically. Some novel treatment strategies for osteoporosis have been developed previously^2^. A prominent example is denosumab, which is a monoclonal antibody for RANKL that aims to inhibit excessive bone resorption. We show that GDF11 is a strong stimulator of RANKL-induced osteoclast formation. Therefore, GDF11 inhibition might be a potential new approach for osteoporosis treatment, especially in patients with high levels of GDF11.

In summary, we show that GDF11 treatment leads to bone loss in both young and aged mice. We further show that GDF11 stimulates RANKL-induced osteoclast formation *in vitro*. This effect is mainly through increased phosphorylation of Smad2/3 and c-Fos, which subsequently stimulates the transcription of *Nfatc1*. GDF11 also inhibits osteoblast differentiation by Smad2/3-dependent repression of Runx2. In addition, rGDF11 treatment impairs bone regeneration in both young and aged mice, and blocking GDF11 function prevents oestrogen-deficiency-induced bone loss and ameliorates age-related osteoporosis. Our data demonstrate that GDF11 is a critical regulator of bone remodelling, and suggest that GDF11 may be a potential target for treatment of osteoporosis.

## Methods

### Animals

Female C57BL mice were obtained from the Experimental Animal Center of Sichuan University and housed in pathogen-free facilities under a 12-h light and 12-h dark cycle. All protocols were approved by the Subcommittee on Research and Animal Care (SRAC) of Sichuan University.

### MicroCT analysis

MicroCT analysis was performed according to recent guidelines^56^ using a Skyscan 1176 microCT imaging system (Skyscan, Kontich, Belgium) with a spatial resolution of 12 μm (X-ray source 50 kV/455 kA; exposure time 0.265 s; magnification × 15; 1 mm filter applied). Volumetric reconstructions and analyses were performed using built-in software NRecon 1.6 and CTAn 1.8, *respectively*. For the analysis of bone regeneration, the volume of interest was defined as a cylindrical area covering the initial bone defect. Bone volume (BV/TV, %) was calculated within the delimited volume of interest.

### Bone histology and histomorphometry

Processing of undecalcified bone specimens and cancellous bone histomorphometry were performed as described previously^57^. Femurs and vertebrae were fixed in 10% buffered formalin overnight and then stored in 70% ethanol at 4 °C before being processed and embedded in methylmethacrylate. Five-μm-thick sections were prepared using a Leica RM2235 microtome and were stained by the von Kossa/nuclear fast red method. Histomorphometric measurements in the distal femur and L4 vertebrae were made using OsteoMeasure software (OsteoMetrics, Decatur, GA). All histomorphometric parameters were calculated and expressed according to the suggestions made by the ASBMR nomenclature committee^58^.

### ELISA

Serum concentrations of CTX and P1NP were measured using commercial ELISA kits from IDS (Fountain Hills, AZ). Blood was collected 4 weeks after rGDF11 treatment or 2 weeks after GDF11 Ab treatment by puncturing the cheek pouch of animals. Before blood collection, the mice were fasted for 4 h.

### Cell culture

We cultured the cells in minimum essential medium α (α-MEM) supplemented with 10% foetal bovine serum, 100 units ml^−1^ penicillin, 100 μg ml^−1^ streptomycin and 10 mM HEPES (all from Gibco). Mouse BMMs were cultured as follows. Tibiae of 6–9-week-old mice were aseptically removed and the bone marrow cells were flushed out. The cells were suspended and cultured in the medium containing 100 ng ml^−1^ macrophage colony-stimulating factor (M-CSF) (416-ML/CF, R&D, Minneapolis, MN) overnight at 37 °C. The untouched cells were collected and further cultured with 100 ng ml^−1^ M-CSF for 2 days to obtain BMMs. The resultant BMMs were then differentiated into osteoclasts in the presence of 100 ng ml^−1^ M-CSF and 50 ng ml^−1^ RANKL (462-TR/CF, R&D, Minneapolis, MN) with or without rGDF11 (#120-11, PeproTech, Rocky Hill, NJ) for 4 days. Subsequently, cells were fixed and stained using TRAP for evaluation. We counted TRAP-positive cells with at least three nuclei as osteoclasts. For the resorption assay, BMMs were seeded on an Osseo Assay surface (Corning) and differentiated into osteoclasts. After 7 days, the cells were removed by incubation with 10% bleach solution for 10 min, and the resorption pits were visualized by phase microscopy.

BMSCs were isolated from 9-week-old mice by flushing the bone marrow of tibiae and femurs^59^. Primary osteoblasts were isolated from the calvariae of 3-day-old mice as described previously^60^. Briefly, calvariae were dissected aseptically, and sequentially digested with 1 mg ml^−1^ collagenase solution containing collagenase types I and II in 1:3 ratio (Worthington, Newark, NJ). Osteoblast enriched fractions (fraction 3 to 6) were collected and cultured for around 7 days until confluence. Osteoblast differentiation was induced by treating cells with medium containing 50 μM ascorbic acid, 10 mM β-glycerophosphate and 10 nM dexamethasone (all from Sigma). For co-culture assay, BMMs were seeded on the confluent osteoblast layer and cultured for 5 days in presence of 10 nM 1,25-dihydroxyvitamin D_3_ (1,25(OH)_2_D_3_, Sigma) and 1 μM prostaglandin E_2_ (PGE_2_, Sigma) with or without 100 ng ml^−1^ rGDF11.

### Cell sorting and colony formation assay

We collected the bone marrow cells from the tibiae of rGDF11- or vehicle-treated animals by flushing. Cells were incubated in the red blood cell lysis buffer for 5 min at room temperature. The sorting of BMSCs were performed as described^61^. Briefly, the cell aliquots were incubated with phycoerythrin (PE)-, fluorescein isothiocyanate (FITC)-, peridinin chlorophyll protein (Per CP)-, and allophycocyanin (APC)-conjugated antibodies against mouse CD29, Sca-1, CD11b and CD45 (BioLegend, San Diego, CA) at 4 °C for 30 min. Cell sorting was performed on a fluorescence-activated cell sorting (FACS) Aria model (BD Biosciences), and analysis was performed with fluorescence-activated cell sorting DIVE software version 6.1.3 (BD Biosciences).

The sorted Sca-1^+^CD29^+^CD45^−^CD11b^−^ cells were collected and plated into 6-well plates (1,000 cells per well). After 4 h of adhesion, unattached cells were removed. Medium was changed every 3 days, and cultures were fixed and stained with 0.5 mg ml^−1^ of crystal violet on day 10. Colonies containing 50 or more cells were counted.

### siRNA-mediated knockdown

Mouse c-Fos and control small interfere RNAs (siRNAs) were purchased from Santa Cruz (Dallas, Texas). Each siRNA consists of pools with three to five target-specific 19–25-nucleotide siRNAs designed to knockdown target gene expression. For siRNA-mediated knockdown, mouse BMMs were transfected using Lipofectamine RNAimax (Invitrogen) following the manufacturer's instruction.

### Proliferation and apoptosis assay

The proliferation of BMMs, BMSCs and osteoblasts was determined using Cell Proliferation Reagent WST-1 (Roche, Indianapolis, IN). Briefly, 10 μl of reagent was added to each well, including three wells containing only medium for background subtraction. After incubation at 37 °C for 1 h, the absorbance at 450 nm was measured using a microplate reader (Varioskan Flash, ThermoScientific). The apoptosis assay of BMMs was performed using an *in situ* Cell Death Detection kit (Roche) according to the manufacturer's instruction.

### Microarray analysis and quantitative RT-PCR

Expression levels of messenger RNA were measured by microarray analysis of mouse BMMs 24 h after stimulation in the presence of 100 ng ml^−1^ M-CSF and 50 ng ml^−1^ RANKL with or without 100 ng ml^−1^ rGDF11. Total RNA was isolated using an RNeasy kit (Qiagen) according to the manufacturer's protocol. Microarray experiments were performed in triplicate using the Affymetrix Mouse Gene 1.0 ST Array. The robust multichip average method was used to normalize the gene expression raw data. We calculated the gene expression levels using Affymetrix Expression Console and Transcriptome Analysis Console 3.0 software. The threshold for differentially regulated transcripts was set as the fold change of 1.5 with a *P* value of <0.01.

For quantitative RT-PCR, cDNA was prepared from 2 μg RNA using a QuantiTec reverse transcription kit (Qiagen) and analysed with SYBR GreenMaster Mix (SABiosciences) in ABI7500 real-time PCR system (Applied Biosystems, Foster City, CA). The primers designed for each targeted gene are listed in Supplementary table 1. Relative expression was calculated using a 2^−ΔΔCt^ method by normalizing with *Gapdh* housekeeping gene expression and presented as fold increase relative to control.

### Western blot

Cells were lysed in RIPA buffer (Pierce, Rockford, IL) on ice. The samples were denatured at 95 °C for 5 min in sample buffer containing 2% SDS and 1% 2-mercaptoethanol, separated on 10 or 15% SDS–polyacrylamide gels, and transferred to polyvinylidene difluoride membranes by a semi-dry transfer apparatus (Bio-Rad, Hercules, CA). For native condition, rGDF11 or Myostatin (#120-00, PeproTech, Rocky Hill, NJ) were added into the loading buffer (62.5 mM Tris-HCl, pH 6.8; 40% glycerol; and 0.01% Bromophenol Blue), and electrophoresed on 4–15% Mini-PROTEAN TGX gels (Bio-Rad, Hercules, CA) in a running buffer containing 25 mM Tris Base and 192 mM glycine at 4 °C. The proteins were then transferred to polyvinylidene difluoride membranes at 4 °C by a wet transfer apparatus (Bio-Rad, Hercules, CA). The membranes were blotted with 5% milk for 1 h and then incubated with primary antibodies overnight. The following antibodies were used: mouse anti-GDF11 (0.5 μg ml^−1^, MAB19581, clone #743833, R&D), rabbit anti-phospho-Smad2 (1:1,000, MA5-15122, l, ThermoFisher), rabbit anti-phospho-Smad3 (1:1,000, #9520, Cell Signaling), rabbit anti-phospho-Smad2/3 (1:1,000, #8828, Cell Signaling), rabbit anti-Smad2/3 (1:1,000, #8685, Cell Signaling), rabbit anti-phospho-c-Fos (1:1,000, #5348, Cell Signaling), rabbit anti-c-Fos (1:1,000, #2250, Cell Signaling), mouse anti-NFATc1 (1:2,000, MA3-024, ThermoFisher), rabbit anti-phospho-Smad1/5 (1:1,000, #9516, Cell Signaling), and rabbit anti-Smad1/5/8 (1:1,000, SC-6031-R, Santa Cruz). The immunocomplexes were incubated with horseradish peroxidase conjugated anti-rabbit or anti-mouse IgG (Jackson ImmunoResearch, West Grove, PA) and visualized with SuperSignal reagents (Pierce, Rockford, IL). The ImageJ software was used for densitometric analyses of the blots, and the quantification results were normalized to the loading control.

### Chromatin Immunoprecipitation (ChIP) assay

The antibodies for ChIP were as follows: acetylated histone H4 (H4ac, #39926, Active Motif), Smad2/3 (#8685, Cell Signaling) and c-Fos (#2250, Cell Signaling). ChIP assays were performed using a SimpleChIP Assay Kit (Cell Signaling Technology) according to the manufacturer's protocol. Briefly, cells were incubated in 5 mM dimethyl 3,3′-dithiobispropionimidate-HCl (DTBP; Pierce, Rockford, IL) for 10 min at room temperature, rinsed with 100 mM Tris-HCl buffered saline (pH 8.0) and crosslinked with 1% formaldehyde in PBS for 10 min at 37 °C. Precipitated DNA samples were quantified with real-time PCR. Data are presented as the percentage of input DNA. The primers are listed in Supplementary table 1.

### Immunohistochemical staining

The dissected femurs were fixed with 10% buffered formalin overnight and decalcified with 15% EDTA in a cold room for 2 weeks before processing for paraffin sections. Sections were treated in sodium citrate buffer (10 mM Sodium citrate, 0.05% Tween 20, pH 6.0) at 90 °C for 20 min for antigen retrieval and incubated with rabbit anti-phospho-Smad2/3 (1:100, #8828, Cell Signaling; or sc-11769, Santa Cruz), rabbit anti-phospho-c-Fos (1:200, #5348, Cell Signaling) or mouse anti-NFATc1 (1:100, MA3-024, ThermoFisher) antibodies overnight at 4 °C. The DAKO Envision+ HRP (AEC) system was used to detect primary antibodies. We quantified the integral optical density (IOD) of positive staining using Image Pro Plus 6.0 (MediaCybernetics) and normalized by stained area.

### ALP and mineralization assay

BMSCs and primary calvarial osteoblasts were grown in osteogenic differentiation medium supplemented with or without 100 ng ml^−1^ rGDF11. On day 7, cells were fixed with 3.7% formaldehyde and incubated with a staining solution of 0.25% naphthol AS-BI phosphate and 0.75% Fast Blue BB dissolved in 0.1 M Tris buffer (pH 9.3). ALP activity was also quantified using a commercial kit according to the manufacturer's protocol (Cell Biolab, San Diego, CA).

For mineralization assays, cells were cultured in differentiation medium for 2 weeks, fixed with 3.7% formaldehyde and stained with 1% Alizarin red S (pH 4.2, Sigma-Aldrich) for 10 min. Mineralized bone nodules stained with alizarin red were destained with 10% cetylpyridinium chloride in 10 mM sodium phosphate (pH 7.0), and the calcium concentration was determined by absorbance measurements at 562 nm using a standard calcium curve in the same solution.

### Surgeries

Young (9 week old) and aged (18 month old) female mice were anaesthetized with intraperitoneal injection of a combination of ketamine (100 mg kg^−1^) and xylazine (10 mg kg^−1^). In addition, buprenorphine (0.05 mg kg^−1^) was given for perioperative analgesia to minimize suffering and pain. Femoral cortical bone defects were created as described^34^,^62^,^63^. Briefly, the medial surfaces of the bilateral femurs (distal portion) were exposed by blunt dissection of the quadriceps after skin incision. A 1.0 mm hole was generated using a round bur (Komet, Germany) operating at 10,000 r.p.m. under saline irrigation.

Subcritical-sized cranial defects were created following the published surgical protocols^62^,^64^. After careful exposure of the flat surface of the cranium via skin incision, two 1.0 mm symmetrical full-thickness bone defects were generated across the sagittal suture using a cylindrical low-speed carbide bur (Komet, Germany). Periosteum was closed using interrupted 5-0 Monocryl sutures before skin closure.

For bilateral ovariectomy (OVX), the ovaries of 9-week-old animals were approached through a midline dorsal skin incision and exposed by cutting through the muscle layer. Both ovaries were removed after ligating the uterine horn, and the incision of the muscle was sutured with 6-0 silk. The skin was closed with surgical clips. Sham surgery included anaesthesia, midline dorsal incision, exteriorizing and returning of the ovary, and closure of the wound. Antibody treatment started 2 days after the surgery.

### Statistical analysis

All values are expressed as mean±s.d. Statistically significant differences were evaluated by two-tailed Student's *t* test for comparison between two groups or by one-way analysis of variance followed by the Tukey's test for multiple comparisons. A *P* value of <0.05 was considered to be statistically significant.

### Data availability

Microarray data that support the findings of this study have been deposited in NCBI GEO with the primary accession code GSE84609. The authors declare that all other data supporting the findings of this study are available within the article and its Supplementary information files.

## Additional Information

**How to cite this article:** Liu, W. *et al*. GDF11 decreases bone mass by stimulating osteoclastogenesis and inhibiting osteoblast differentiation. *Nat. Commun.* 7:12794 doi: 10.1038/ncomms12794 (2016).

## Supplementary Material

Supplementary Information Supplementary Figures 1-4 and Supplementary Table 1
