# Supplementary material for: GDF11 decreases bone mass by stimulating osteoclastogenesis and inhibiting osteoblast differentiation
Source: Nat Commun. 2016 Sep 22;7:12794. doi: 10.1038/ncomms12794 (PMC5036163; doi:10.1038/ncomms12794)
Supplement: Supplementary Information — Supplementary Figures 1-4 and Supplementary Table 1 [file ncomms12794-s1.pdf]

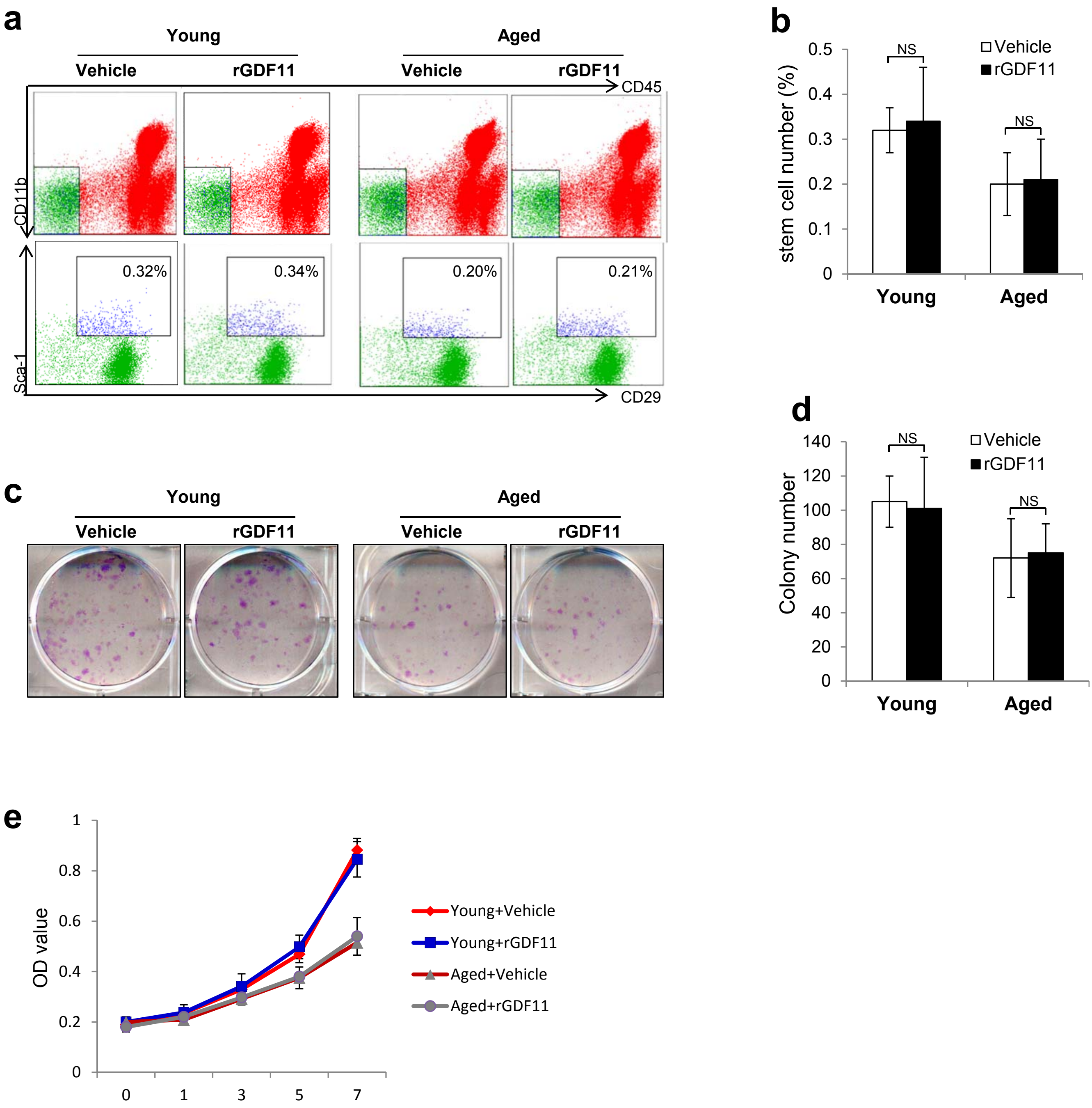

Supplementary Figure 1. GDF11 treatment did not rejuvenate the BMSCs. (a, b) Flow cytometry analysis of the bone marrow cells. (c, d) Colony formation assay of the sorted BMSCs. (e) Proliferation assay of the sorted BMSCs. Results are shown as mean ± SD; n=6; NS: no significance by *t* test.

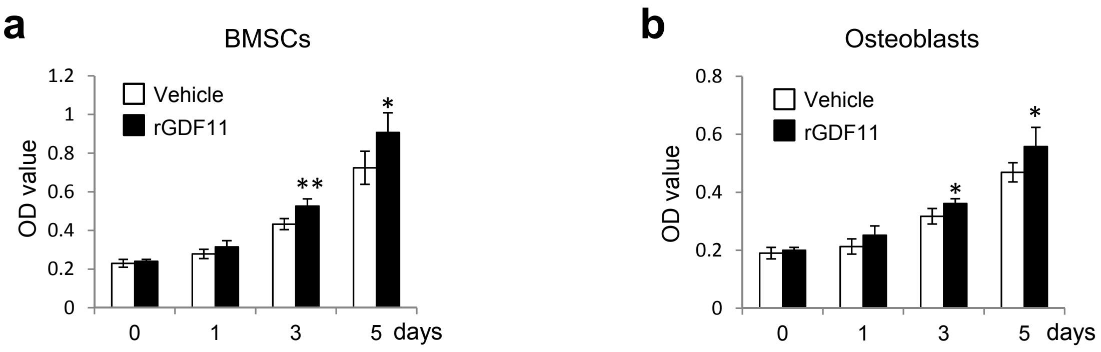

Supplementary Figure 2. Proliferation assay. GDF11 slightly increased the proliferation of both BMSCs (a) and primary calvarial osteoblasts (b). Cells were cultured in osteogenic differentiation medium. Results are shown as mean ± SD; n=5; *: p<0.05 and **: p<0.01 by t test.

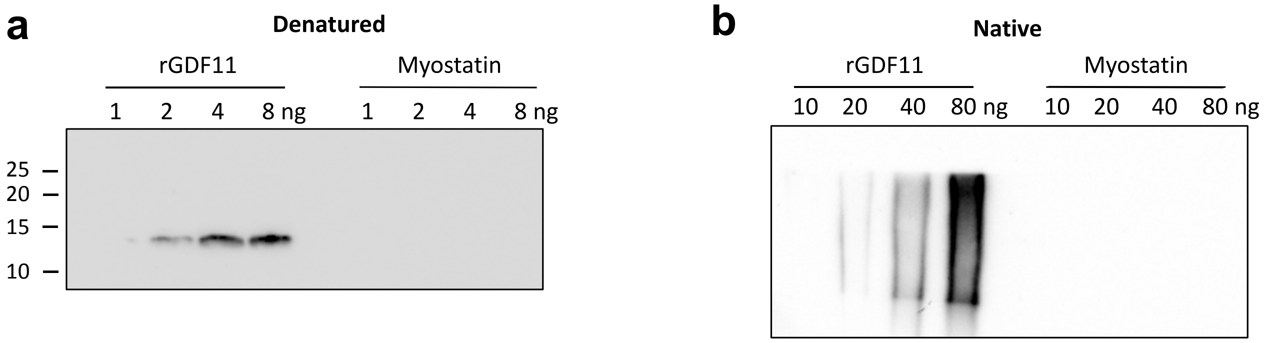

Supplementary Figure 3. Western blot analysis using GDF11 antibody. (a) Denatured Western blot. The proteins were denatured at 95℃ for 5 minutes in sample buffer containing 2% SDS and 1% 2-mercaptoethanol. (b) Native Western blot. The R&D GDF11 antibody (MAB19581, clone #743833) binds specifically to GDF11 without cross reaction with myostatin.

Fig. 4f

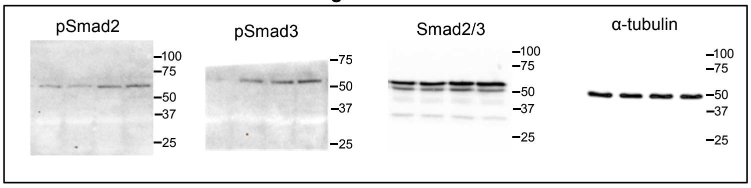

Fig. 4g

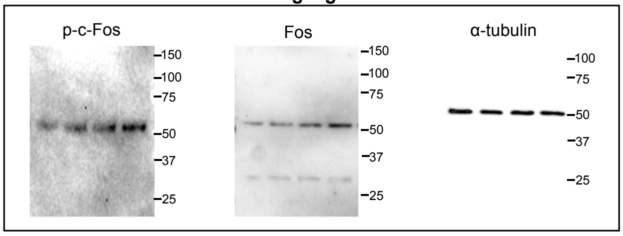

Fig. 4i

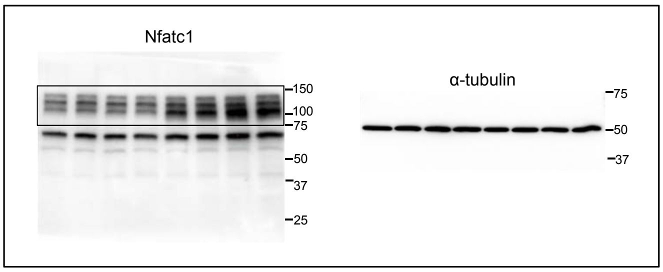

Fig. 4k

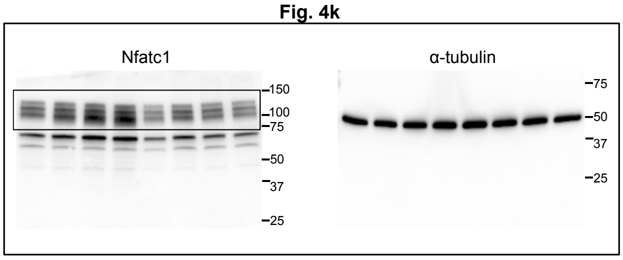

Fig. 5i

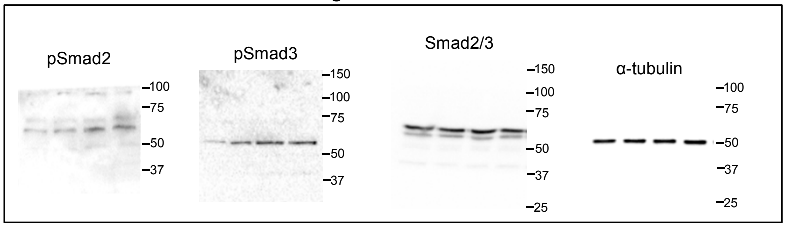

Fig. 5k

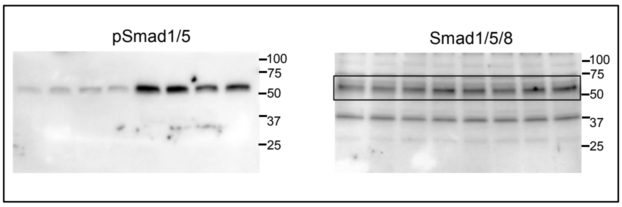

Fig. 5l

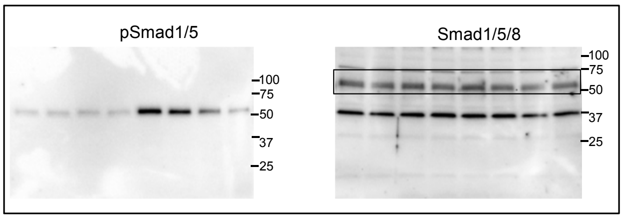

Supplementary Figure 4. Uncropped images of immunoblots.

Supplementary table 1. Primers for quantitative RT-PCR and ChIP assay.

**RT-PCR primers**	
Nfatc1 F	GGAGAGTCCGAGAATCGAGAT
Nfatc1 R	TTGCAGCTAGGAAGTACGTCT
Fos F	CGGGTTTCAACGCCGACTA
Fos R	TTGGCACTAGAGACGGACAGA
Src F	GAACCCGAGAGGGACCTTC
Src R	GAGGCAGTAGGCACCTTTTGT
Acp5 F	CACTCCCACCCTGAGATTTGT
Acp5 R	CATCGTCTGCACGGTTCTG
Ctsk F	GAAGAAGACTCACCAGAAGCAG
Ctsk R	TCCAGGTTATGGGCAGAGATT
Runx2 F	GACTGTGGTTACCGTCATGGC
Runx2 R	ACTTGGTTTTTCATAACAGCGGA
Osx F	ATGGCGTCCTCTCTGCTTG
Osx R	TGAAAGGTCAGCGTATGGCTT
Alp F	CACGGCCATCCTATATGGTAA
Alp R	GGGCCTGGTAGTTGTTGTGA
Ocn F	CTGACCTCACAGATCCCAAGC
Ocn R	TGGTCTGATAGCTCGTCACAAG
Gapdh F	TCATTGACCTCAACTACATG
Gapdh R	TCGCTCCTGGAAGATGGTGAT
**ChIP primers**	
Nfatc1 F	GACTTTGTGTCCCCAGGAGG
Nfatc1 R	GCAGAGGCAGAACACTTCCA
Nfatc1 negative F (20kb down)	GTGCTGTGTGTTATGGCACC
Nfatc1 negative R (20kb down)	CCTGGGGGCAGTGTTTACAG
Ocn F	CTGAGCACATGACCCCCAAT
Ocn R	TCGGCTACTCTGTGCTCTCT
Ocn negative F （2kb down）	AGAAGGCAGGTGTGTTGAGG
Ocn negative R （2kb down）	TGCTCTTCCTGCCTCTACCT
